# Utilization of Matrix Effect for Enhancing Resolution in Cation Exchange Chromatography

**DOI:** 10.3390/molecules29153637

**Published:** 2024-07-31

**Authors:** Boglárka Páll, Róbert Kormány, Krisztián Horváth

**Affiliations:** 1 Laboratory of Drug Substance Analytical Development 1, Egis Pharmaceuticals Plc., Keresztúri út 30-38, H-1106 Budapest, Hungary; pall.boglarka@egis.hu; 2 Laboratory of Structural Chemistry, Egis Pharmaceuticals Plc., Keresztúri út 30-38, H-1106 Budapest, Hungary; kormany.robert@egis.hu; 3 Analytical Chemistry Research Group, University of Pannonia, Egyetem utca 10, H-8200 Veszprém, Hungary

**Keywords:** matrix effect, ammonium hydroxide, tris, sodium

## Abstract

In ion chromatography studies, the matrix effect of other inorganic ions present in the sample is a well-known phenomenon. In this work, the behavior of inorganic and organic ions was studied in a system overloaded with ammonium ions. The ammonium ions came from a solution of ammonium hydroxide in various concentrations (0.25–1.25%). In this system, which was significantly overloaded with ammonium ions, the behavior of three ions were tested (lithium, tris, and sodium cations). The measurements were performed at different eluent concentrations (6–17 mM), chromatographic column temperatures (25–40 °C), and injected volumes (15–40 µL). The retention times of sodium and lithium ions increased with increasing amounts of injected ammonium, while tris remained essentially unchanged, indicating that the resolution of these ions can be influenced by varying the concentration of the matrix. The results suggested that the observed effect was due to a combination of the pH change caused by the injected matrix, the dissociation of tris ions, the dissociation of the carbocylic ion-exchange groups of stationary phase, the change in buffer capacity, and the amount of ammonium ion introduced. It has been shown that in a well-designed experiment, the addition of ammonium hydroxide to the sample at concentrations greater than 1% can improve the efficiency of organic and inorganic cation separation. It was found that 8 mM methanesulfonic acid eluent, 30 °C, 1% ammonium hydroxide matrix concentration, and 25 µL injection were optimal for the baseline separation of tris and sodium ions on the high-capacity Dionex CS16 column. These ions could not be separated on this column without the presence of the ammonium matrix.

## 1. Introduction

Matrix effects are harmful to separation in most cases and should be prevented. In ion chromatography, the matrix effect could be caused by the eluent (by affecting detection) or by the sample analyzed (by affecting resolution and detection). At first, the eluent matrix effect can be reduced by using a suppressor, where the ions of the eluent are transformed to water, thus reducing the conductivity of the eluent and increasing the sensitivity of the measurement [1]. An analysis of samples with large amounts of ions is a difficult task, because the overloaded system makes it impossible to determine the solute ions. The high ion concentration in the sample has an effect on the elution and retention behavior of the ions analyzed. The self-elution effect of the matrix ion causes shorter retention times of the other ions, while the lower affinity of the interfering ion for the stationary phase may cause a delayed retention time of the analyte ions [2], and the retaining of retention time depends on the concentration of the matrix ion [3,4].

Krzysztof Kulisa [5] et al. studied the effect of column overloading by three anions and four cations. The effect of the overloading of each ion on the retention time of the other ions and on the system suitability parameters was investigated individually. It was found that in a system overloaded with any ion, the retention time of the other ions was reduced. The changing retention time under the measurement makes the qualitative analysis more difficult. The solution to this problem could be to use an internal standard, which behaves in a similar way as the analyte ion. It may even be helpful to introduce the matrix ion (if known) into the reference solution at the same concentration as is present in the sample solution.

In some cases, the matrix ion concentration is so high that it deforms or splits the shape of inorganic peaks [3,6]. In addition to the effect of matrix ions, the pH of the analyte has a significant effect on the conductivity of the tested organic anions [7]. In some simple cases, matrix effects can be avoided by changing the detector, e.g., using UV absorption for the analysis of nitrite and nitrate in the presence of high levels of chloride [4,8]. In addition to this, several solutions have been developed to remove the harmful matrix substances by sample preparation, off-line or on-line equipment in the ion chromatography device [9,10], or by switching the types of stationary phases [11].

Several studies present and analyze the matrix effect in suppressed ion chromatography systems, both from the anion exchange and cation exchange side, including some on retention prediction with a large amount of matrix. These predictions are based on equilibrium-based approach and theoretical calculations [12].

In this paper, we demonstrate how the matrix effect can be utilized, rather than avoided, to improve the chromatographic resolution of tris and sodium ions. By varying the concentration of ammonium hydroxide in the sample from 0.25% to 1.25% (∼11 pH), retention times and resolution can be optimized for tris and sodium cations on a Dionex CS16 column. In our work, we investigate a number of variables that can influence this phenomenon, such as eluent concentration, column temperature, and injected volume.

## 2. Materials and Methods

### 2.1. Materials and Equipment

Dionex ICS 5000 HPIC system with EGC (methanesulfonic acid, MSA, Eluent Generator Cartridge, Thermo Scientific, Waltham, MA, USA) and a suppressed conductivity detector (CD) was used for the ion chromatography measurement (Thermo Scientific, Waltham, MA, USA). The cation exchange column was a Dionex IonPac CS16 (3 × 250 mm) with a guard column CG16 (3 × 50 mm) (Thermo Scientific, Waltham, MA, USA). The chromatograms were processed by using Chromeleon 7, Dionex Version 7.1.3.2425 (Thermo Scientific, Waltham, MA, USA). The flow rate of the eluent was set to 0.38 mL/min.

Mettler Toledo analytical and precision balances were used for weighing (Mettler Toledo, Greifensee, Switzerland). Eppendorf automatic pipettes were used for liquid handling (Eppendorf SE, Hamburg, Germany). The ammonium hydroxide solution, 25% (Merck KGaA, Darmstadt, Germany), lithium chloride, sodium chloride, and tris (Sigma-Aldrich, Darmstadt, Germany) used were of analytical grade. Purified water was prepared freshly using an ELGA Purelab system (ELGA, Lane End, UK).

### 2.2. Method

The investigated matrix ion in our case is ammonium ion, so tests were made to observe its effect for sodium, tris, and lithium ion retention behavior. The samples were ammonium hydroxide solutions (diluted from 25% ammonium hydroxide solution) with added lithium (concentration: 0.6 mg/L), sodium (concentration: 0.6 mg/L), and tris (concentration: 40 mg/L) cations. The column temperature varied between 25 and 40 °C, the methanesulfonic acid eluent concentration varied between 6 and 17 mM, the injected volume varied between 15 and 40 µL, and the ammonium hydroxide concentration varied between 0.25 and 1.25% (m/m). A high-capacity (8400 µq/column) cation exchange column was used for the measurements. The Dionex IonPac CS16 with a CG16 guard column has a 5 µm bead diameter, and its higher capacity helps to improve the performance parameters of cations in the presence of high amounts of ammonium and sodium ions [13].

The investigated concentration range of ammonium ion was 800–4000 mg/L according to the calibration curve and the measured NH_4_OH solutions’ peak areas. In this range, the calibration curve of the ammonium ion is out of the linearity, in a highly overloaded range.

## 3. Results

### 3.1. Effect of the Matrix on Retention Times

The effect of ammonium hydroxide as a matrix was studied on the retention times of sodium ion. The results are presented in Table 1. Contrary to our expectations, retention times increased significantly. At a 1.25% NH_4_OH matrix concentration, a nearly 10% (1.6 min) increase of retention time was experienced. This is opposite to what other authors have observed in similar ion chromatography systems (e.g., Krzysztof Kulisa et al. [5]). In the following, this effect is investigated in a thorough and systematic way. In addition, the matrix effect will be used to enhance the resolution of organic and inorganic anions.

### 3.2. Effect of the Eluent Concentration

**Figure 1:**
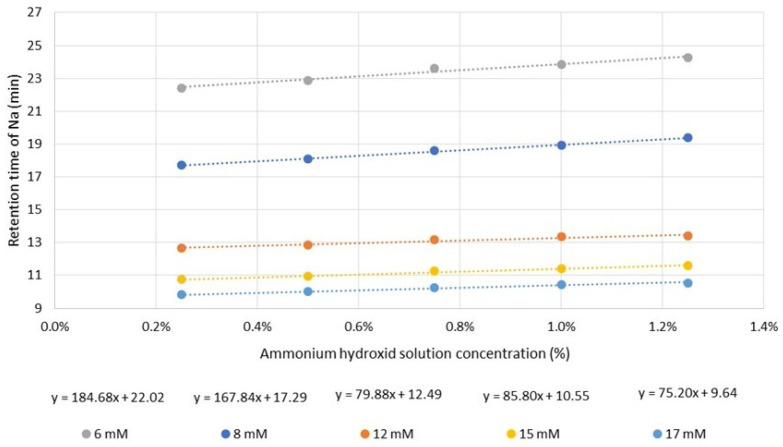
The retention times of sodium ion by varying the ammonium hydroxide solution concentration and the eluent (methanesulfonic acid, MSA) concentration.

The effect of varying eluent concentration on the retention shift of sodium ion was investigated. The results obtained can be well approximated by linear equations, shown in Figure 1. The slopes of the fitted linear curves shows that the matrix effect is more significant for low eluent concentrations. At lower eluent concentrations, the migration velocity of solute is lower. Consequently, it travels together with the plug of matrix ions inside the column for a longer time, resulting in an increased matrix effect. Additionally, as the eluent concentration decreases, the relative concentration of ammonium hydroxide increases. This has a dual impact; it significantly alters the elution power and also the eluent pH.

**Figure 2:**
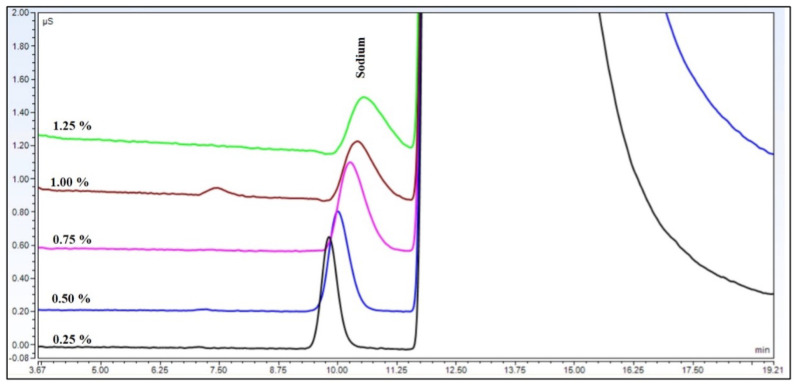
Peaks of sodium cations at different levels of ammonium hydroxide matrix eluted with 17 mM methansulfonic eluent at 40 °C.

**Figure 3:**
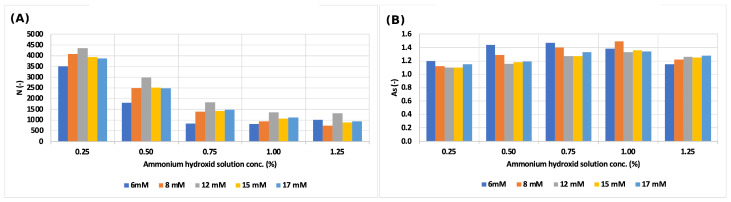
(**A**) Change in the theoretical plate number values of sodium ion by varying the ammonium hydroxide solution concentration and the eluent (MSA) concentration; (**B**) change in symmetry factor values of the sodium ion by varying the ammonium hydroxide solution concentration and the eluent (MSA) concentration.

The theoretical plate number of the sodium ion decreased to more than a fifth, but the symmetry factor did not change significantly (Figure 2). The theoretical plate number and symmetry factor values changed in the same way in each eluent concentration, as is shown in Figure 3.

Taking into consideration the degree of matrix effects and the total analysis time, the chosen concentration of the methanesulfonic acid eluent was 8 mM for further experiments.

### 3.3. Effect of Column Temperature

**Figure 4:**
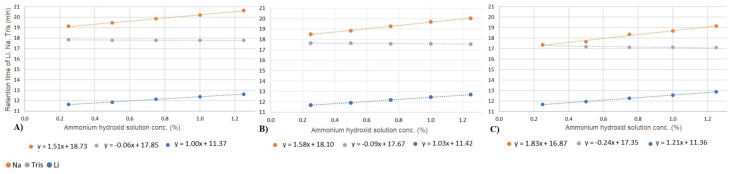
Change in retention time of lithium (blue), tris (gray), and sodium ions (orange) by varying the ammonium hydroxide solution concentration at 25 °C (**A**), 30 °C (**B**), and 40 °C (**C**).

We investigated how changes in column temperature impact various chromatographic parameters, including retention time, plate number, and symmetry. Our study focused on three ions, lithium, sodium, and tris, under varying concentrations of the ammonium hydroxide matrix and across different column temperatures. Figure 4 summarizes the impact of temperature on retention times. Consistent with previous findings (see Section 3.2), our results reveal a linear correlation between ammonium hydroxide concentration and ion retention. However, the effect of increasing ammonium hydroxide concentrations differs for organic and inorganic ions. Specifically, while the retention time of the two inorganic ions increases, the retention time of tris decreases. Notably, the slope of the fitted linear functions indicates that the matrix effect becomes more pronounced at higher temperatures. As is discussed later in Section 3.5, the overall phenomenon is influenced by at least five different simultaneous effects. Each of these processes can be affected by temperature. The effect of temperature cannot be explained precisely; it requires further specific studies.

Figure 4 also reveals that the separation of sodium and tris is not possible at low matrix concentrations. However, due to the distinct behavior of these cations in response to increasing matrix levels, the resolution of the studied cations can be optimized. The resolutions exhibit varying trends among the three ions. While the resolution between lithium and tris decreases, an opposite trend is observed between tris and sodium. As indicated in Table 2, below 40 °C, tris and sodium begin to separate, and at lower temperatures with higher ammonium hydroxide concentrations, the separation becomes more effective. Despite tris experiencing decreased retention at higher temperatures and sodium showing increased retention, the difference in their retention times is negligible at low matrix concentrations. Consequently, the net effect of these opposing factors results in the most favorable separation conditions occurring at 25 or 30 °C with matrix concentrations higher than 1.00%.

### 3.4. Effect of the Injection Volume

**Figure 5:**
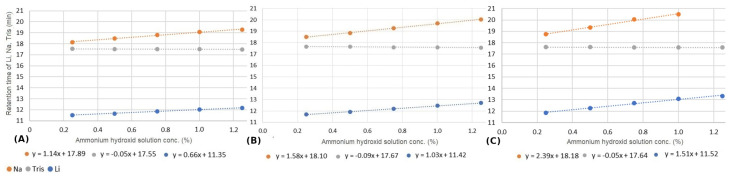
Change in retention time of lithium (blue), tris (gray), and sodium ions (orange) by varying the ammonium hydroxide solution concentration by injecting 15 µL (**A**), 25 µL (**B**), and 40 µL (**C**).

Figure 5 shows the impact of different injection volumes on the retention time of the three ions at different levels of the ammonium hydroxide matrix. These measurements were carried out at 30 °C. Since higher injection volumes also imply higher overloads, we found, not surprisingly, that the matrix effect is more significant at larger injection volumes.

When examining the resolution, we observed that increasing the injected volume leads to improved resolution between tris and sodium cations and decreased resolution between tris and lithium cations (as shown in Table 3). The reason for this phenomenon is that the retention of inorganic cations increases with increasing matrix levels, while the retention of tris remains practically unchanged. As a result, the peak of lithium, which has a lower retention than tris, becomes closer and closer to tris, so that their resolution decreases. On the other hand, the peak of sodium, which has a higher retention than tris, moves further away from the peak of tris, so their resolution increases. However, at 40 µL of injection, the peak shape significantly deteriorates, resulting in poorer values for both the theoretical plate number and the symmetry factor. Consequently, an optimal choice for the separation of Tris and Na^+^ ions was 25 µL.

### 3.5. Study of Matrix Processes

**Figure 6:**
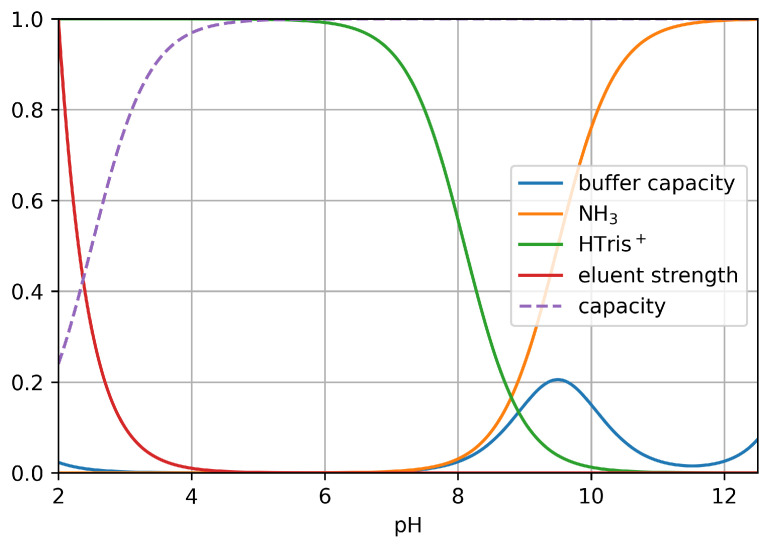
Parameters affected by the alkaline matrix: buffer capacity of injected NH_4_OH matrix (at 1.25%). Orange: molar fraction of dissociated (neutral) NH_3_; green: molar fraction of protonated (cationic) tris; red: relative eluent strength; dashed purple: relative ion-exchange capacity (assumed pKa: 2.5).

In the system studied, the matrix influences solute retention through different concurrent processes that take place simultaneously (also see Figure 6), listed as follows:

1. Weakening eluent
  The separation takes place under acidic conditions in the system studied. H_3_O^+^ cations compete with positively charged sample ions for functional groups on the stationary phase in ion-exchange equilibria. When the alkaline sample is injected into the column, the local pH becomes alkaline. As the concentration of H_3_O^+^ cations decreases, the local retention factor of solutes increases. Consequently, sample compounds may become completely trapped at the head of the column for a certain period of time. Accordingly, this effect increases the retention of all analytes.
2. Protonation/dissociation of tris
  In the system studied, under acidic elution and neutral detection conditions, tris exists in its protonated, cationic form. However, at the alkaline pH of the injected sample, tris dissociates, resulting in a neutral molecule that does not retain on the ion-exchange functional groups. This phenomenon leads to a reduced retention of tris.
3. Self-eluting effect and protonation of ${\mathrm{NH}}_{4}^{+}$ matrix ions
  The high concentration of injected ammonium cations competes with other sample cations in the ion-exchange equilibria, resulting in reduced retention factors. This effect is called self-elution [12]. However, at the highly alkaline pH of the injected sample, the matrix mostly exists in the neutral form of ammonia, which does not exhibit any self-elution effect. As the eluent’s pH decreases during the elution, the amount of ammonium cation increases, leading to the decreased retention of solutes. However, due to the dissociation of ammonium ions, this decrease is not as significant as it would be with a simple cation matrix.
4. Buffer capacity of the matrix
  The opposing effects of the processes listed above are further influenced by the significant buffering capacity of the injected matrix. As a result, the eluent’s pH returns slightly more slowly to the acidic range than expected. All of this somewhat enhances the impact of the aforementioned processes compared to what would have occurred if the matrix had been a strong base.
5. Ion-exchange capacity of the weak cation exchanger
  The stationary phase used in this work is a weak cation exchanger. Accordingly, its ion-exchange capacity is reduced at acidic pHs, while at basic pHs, its capacity reaches its maximum. By the injection of the alkaline sample, the local ion-exchange capacity of the stationary phase increases temporarily, increasing the retention of cations. Since tris is deprotonated at the pH of injection, this effect is significantly lower for tris than for the other two inorganic cations.
  It should be noted that the pKa of the CS16 phase is not publicly available. Normally, the pKa of the -COOH group is around 4–5, but this can be significantly altered by adding substituents. Since this phase is generally used with acidic eluents at pHs 1.5–2, we can assume that the acidity of the carboxyl groups has been adjusted by the manufacturer. For simplicity, in Figure 6, a value of 2.5 pKa was assumed. Even if this is an assumption, the quantitative description of the matrix effect is still valid.

**Figure 7:**
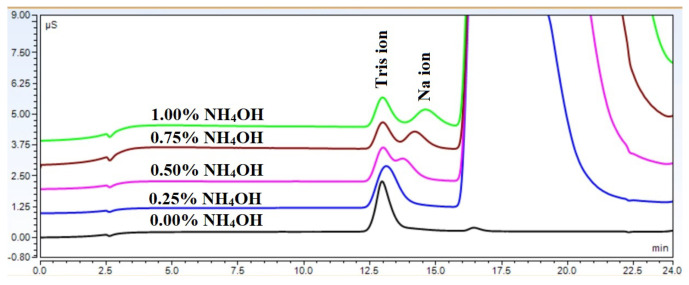
Effect of increasing ammonium hydroxide concentration on the resolution between sodium and tris.

The retention profiles shown in Figure 1, Figure 4, and Figure 5 are the net result of these counteracting processes. However, these effects can be utilized to improve the resolution of closely eluting compounds, as shown in Figure 7. It can be seen that sodium and tris could not be separated without the presence of the ammonium hydroxide matrix. However, as the sample matrix content increased, the separation became more and more pronounced. Eventually, even baseline separation became possible.

## 4. Conclusions

The matrix effect is, in most cases, detrimental to sample identification and should be eliminated, but in this work, it has been shown, through an example, that it can be useful. Ammonium hydroxide has an effect on retention times and performance parameters for other ions. These investigated systems are very complex, and the experienced matrix effect is the result of several effects, which are the concentration of the eluent, the buffer capacity of the analyte, and the large amount of ammonium ion. Based on the results, it can be said that by setting the appropriate conditions, the retention time and resolution of the tested inorganic and organic ions (lithium, tris, sodium) can be planned, and by taking advantage of the matrix effect of ammonium hydroxide, the tris cation and the sodium ion can be separated with adequate resolution on a high-capacity cation exchange column (Dionex Ionpac CS16) under properly adjusted conditions. This phenomenon can be useful for the determination of tris, where sodium ion interferes with measurement on a Dionex CS16 column.

## Figures and Tables

**Table 1:** Change in sodium retention time by increasing the ammonium hydroxide concentration at an 8 mM eluent concentration.

NH_4_OH Solution Concentration	Sodium Ion Retention Time	Increase in Sodium Ion Retention Time ^1^
%	(t_*R*_, min)	(t_*R*_, %)
0.25	17.703	
0.50	18.097	2.23
0.75	18.623	5.20
1.00	18.913	6.83
1.25	19.393	9.55

^1^ Compared to the initial value (0.25% NH_4_OH solution concentration.)

**Table 2:** Effect of column temperature on the resolution between the three cations by varying ammonium hydroxide concentration.

	Column Temperature					
	25 °C		30 °C		40 °C	
NH_4_OH Concentration	R_*s*_ [-]	R_*s*_ [-]	R_*s*_ [-]	R_*s*_ [-]	R_*s*_ [-]	R_*s*_ [-]
%	Li-Tris	Tris-Na	Li-Tris	Tris-Na	Li-Tris	Tris-Na
0.25	5.3	-	5.7	-	4.8	-
0.50	4.7	-	5.1	-	4.3	-
0.75	4.1	1.2	4.3	1.1	3.9	-
1.00	3.6	1.3	3.8	1.2	3.0	0.9
1.25	3.1	1.5	3.3	1.3	2.3	1.0

**Table 3:** Effect of injected volume on the resolution between the three cations by varying ammonium hydroxide concentration.

	Injected Volume					
	15 μL		25 μL		40 μL	
NH_4_OH Concentration	R_*s*_ [-]	R_*s*_ [-]	R_*s*_ [-]	R_*s*_ [-]	R_*s*_ [-]	R_*s*_ [-]
%	Li-Tris	Tris-Na	Li-Tris	Tris-Na	Li-Tris	Tris-Na
0.25	6.2	-	5.7	-	5.3	-
0.50	5.7	-	5.1	-	4.1	1.1
0.75	5.0	-	4.3	1.1	3.1	1.2
1.00	4.5	1.1	3.8	1.2	2.4	1.3
1.25	4.1	1.1	3.3	1.3	1.7	-

## Data Availability

Research data are not available from the authors.

## Abbreviations

The following abbreviations are used in this manuscript:

CD	Conductivity Detector
EGC	Eluent Generator Cartridge
HPIC	High-Performance Ion Chromatography
UV	Ultra-Violet

## References

[1] Dasgupta P.K. (1992). Ion chromatography. The state of the art. Anal. Chem..

[2] Neele J., Cleven R., Wiel H. (2002). Matrix Effects in the Determination of Low Anion Concentrations using Suppressed IC. Int. J. Environ. Anal. Chem..

[3] Divjak B., Goessler W., Haddad P., Novič M. (2003). Interference of some matrix ions in cation-exchange chromatography. J. Chromatogr. A.

[4] Michalski R., Lyko A., Kurzyca I. (2012). Matrix Influences on the Determination of Common Ions by using Ion Chromatography Part 1-Determination of Inorganic Anions. J. Chromatogr. Sci..

[5] Kulisa K., Dybczyński R., Polkowska-Motrenko H. (1999). Effect of column overloading and its influence on the quality of analytical results in the determination of inorganic ions by ion chromatography. Chem. Anal..

[6] Novič M., Divjak B., Pihlar B. (1998). On-column processes in ion chromatographic determination of nitrite and nitrate in heavy mineralised samples. J. Chromatogr. A.

[7] Pecyna-Utylska P., Michalski R. (2021). The Influence of Sample pH on the Determination of Selected Carboxylic Acids by Isocratic Ion Chromatography. Chem. Chem. Technol..

[8] Jackson P. (2008). Determination of Inorganic Anions in Wastewater by Ion Chromatography.

[9] Slingsby R., Kiser R. (2001). Sample treatment techniques and methodologies for ion chromatography. TrAC Trends Anal. Chem..

[10] Slingsby R., Pohl C. (1996). Approaches to sample preparation for ion chromatography sulfate precipitation on barium-form ion exchangers. J. Chromatogr. A.

[11] Rey M.A., Riviello J.M., Pohl C.A. (1997). Column switching for difficult cation separations. J. Chromatogr. A.

[12] Hajós P., Horváth K. (2008). Equilibrium-based approach for prediction of matrix-related interferences in anion chromatography. J. Chromatogr. A.

[13] Thomas D., Rey M., Jackson P. (2002). Determination of inorganic cations and ammonium in environmental waters by ion chromatography with a high-capacity cation-exchange column. J. Chromatogr. A.

